# Collaboratively charting the gene-to-phenotype network of human congenital heart defects

**DOI:** 10.1186/gm137

**Published:** 2010-03-01

**Authors:** Roland Barriot, Jeroen Breckpot, Bernard Thienpont, Sylvain Brohée, Steven Van Vooren, Bert Coessens, Leon-Charles Tranchevent, Peter Van Loo, Marc Gewillig, Koenraad Devriendt, Yves Moreau

**Affiliations:** 1 Bioinformatics Group, Department of Electrical Engineering, ESAT-SCD, Katholieke Universiteit Leuven, Kasteelpark Arenberg 10, B-3001 Leuven, Belgium; 2 Université de Toulouse, UPS, Laboratoire de Microbiologie et Génétique Moléculaires, 118 route de Narbonne, F-31000 Toulouse, France; 3 Centre National de la Recherche Scientifique, LMGM, 118 route de Narbonne, F-31000 Toulouse, France; 4 Center for Human Genetics, University Hospital Leuven, Herestraat 49, B-3000 Leuven, Belgium; 5 Laboratory of Molecular Signalling and Laboratory of Developmental Genetics and Imprinting, Babraham Research Campus, Cambridge CB22 3AT, UK; 6 Department of Molecular and Developmental Genetics, VIB, Herestraat 49, box 602, B-3000 Leuven, Belgium; 7 Department of Pediatric Cardiology, University Hospital Leuven, Herestraat 49, B-3000 Leuven, Belgium

## Abstract

**Background:**

How to efficiently integrate the daily practice of molecular biologists, geneticists, and clinicians with the emerging computational strategies from systems biology is still much of an open question.

**Description:**

We built on the recent advances in Wiki-based technologies to develop a collaborative knowledge base and gene prioritization portal aimed at mapping genes and genomic regions, and untangling their relations with corresponding human phenotypes, congenital heart defects (CHDs). This portal is not only an evolving community repository of current knowledge on the genetic basis of CHDs, but also a collaborative environment for the study of candidate genes potentially implicated in CHDs - in particular by integrating recent strategies for the statistical prioritization of candidate genes. It thus serves and connects the broad community that is facing CHDs, ranging from the pediatric cardiologist and clinical geneticist to the basic investigator of cardiogenesis.

**Conclusions:**

This study describes the first specialized portal to collaboratively annotate and analyze gene-phenotype networks. Of broad interest to the biological community, we argue that such portals will play a significant role in systems biology studies of numerous complex biological processes.

CHDWiki is accessible at http://www.esat.kuleuven.be/~bioiuser/chdwiki

## Background

Recently, Wiki technology - inspired by the well-known Wikipedia encyclopedia - has been proposed as a potential strategy for the collaborative development of biological knowledge bases [1-6]. Although a 'Wikipedia for Genes' is likely to emerge, a number of challenges remain. First, classical Wiki technology in itself (based on free text) is unsuitable for developing genetic knowledge bases because of the imperative need for structured information. Hence, Wiki platforms for genetic knowledge bases need to provide a strong framework for integration with classical database technology. Wikiproteins already implements this need at a high level by abstractly linking concepts, such as proteins and biological processes [1]. Second, and probably foremost, each community uses specific terminology, has specific goals, and uses specific data and tools. Such specificity cannot be addressed in a generic Wikipedia for Genes and requires tailored solutions implementing different levels of specialization. Third, Wiki technology does not in itself support downstream analysis of the information gathered in the Wiki.

Going beyond knowledge gathering, integrative data analysis strategies have been proposed recently for the prioritization of genes potentially involved in a given biological process, phenotype, or disease [7-9]. Nevertheless, there is clearly a gap between such advanced (and somewhat complex) analysis strategies and actual wet lab work. A similar gap can be observed between those strategies and clinical genetics where increasingly complex molecular data need to be interpreted towards the diagnosis of constitutional disorders. To bridge this gap and bring integrative analysis strategies into practice, we integrate a candidate gene prioritization method [9] and browsing of networks of gene interactions [10-12] into the Wiki platform.

We therefore propose to combine Wiki technology, databases of genomic and phenomic information, and data analysis tools into a Wiki portal that supports the needs of a specialized community. In particular, we describe a Wiki portal for the genetic study of congenital heart defects (CHDs), termed CHDWiki. CHDs are the major cause of mortality in newborns in the developed world, but despite this manifest importance, most CHDs still have unknown etiologies. In some instances, specific genetic and environmental factors have been shown to cause CHDs. A review of the etiology of CHDs is available through CHDWiki [13].

The CHDWiki portal focuses on mapping out the gene network leading to human CHD phenotypes. It supports both genetic and molecular biology research that aims at hunting for CHD genes, as well as clinical research that aims at identifying and interpreting genetic aberrations in patients suffering from well-characterized CHDs.

## Construction and content

### Knowledge acquisition

To build a set of most currently known gene-phenotype links, OMIM (Online Mendelian Inheritance in Man) and MEDLINE were manually searched by an expert in the field for genes that are linked with any of 139 relevant cardiac defect phenotypes listed among the internationally used CHD codes from the Association for European Paediatric Cardiology (AEPC). The use of this specialized ontology maximizes the relevance of the collected information to the CHD community and improves the consistency of this information. Relevant genes and mutations were selected and their corresponding cardiac phenotype were manually gathered and described based on the available literature. The level of support for a gene-phenotype link was defined by its incidence and the number of independent publications reporting it. We only considered such links confirmed if at least two reports from independent groups described the incidence of CHD in patients with a mutation to be greater than 1%. Moreover, the support for the link between every single gene mutation and CHD type was further characterized based on the genetic evidence (inheritance and incidence), *in silico *predictions, and the functional studies (*in vitro *analysis and animal models) described in the study.

To build a set of most currently known chromosomal regions linked to CHDs, MEDLINE was searched for imbalances detected by molecular karyotyping, breakpoints of balanced chromosome aberrations or regions implicated in CHDs through linkage studies. These data complement at a much higher resolution the CHD regions identified by Van Karnebeek *et al*. [14], which were based on reported cytogenetically visible chromosomal aberrations.

CHDWiki is conceived to allow straightforward inclusion of published and unpublished data from all collaborators. All clinical (cardiac and non-cardiac) and molecular data are rendered anonymous and collected in a standardized manner, either directly in the CHDWiki database (for genes, translocation breakpoints and linkage regions) or (for well-delineated chromosomal imbalances) using another tool designed for this purpose, CHDBench [15]. Consent for submitting unpublished data from a patient or their legal representative is explicitly required, and was assumed to be obtained for published data that are included. Ethical approval for the incorporation of patient data was obtained from the Ethics Committee KU Leuven (S51093).

### Platform development

CHDWiki is based on the MediaWiki engine initially developed for the Wikipedia project. We implemented a generic extension that allows registering specific components for the management of structured data and for the on-the-fly execution of analysis tools.

The benefits of databases are manifold and become apparent when providing different views on the same data. For instance, it allows providing the detailed list of genes linked to CHDs, as well as the list of CHDs having linked genes. Databases also solve consistency issues; for example, when a link is added or updated between a gene and a specific CHD, both the gene page and the CHD page instantaneously reflect this change. The principles of the generic extensions are the following. Specific components can subscribe to pages so that the Wiki engine executes these components when the page is rendered, or they can explicitly be called from within the page through the use of a specific tag.

For example, to include the list of CHDs linked to *JAG1*, the generic extension first calls the chdsForGenes component with parameter *JAG1 *to retrieve the data in the form of (variable, value) pairs. Second, the extension retrieves the chdsForGeneTemplate layout template, which is stored as Wiki text in a standard Wiki page. Third, it replaces the variables by their actual values. Eventually, the resulting Wiki text is rendered by the MediaWiki engine.

The simplicity of the generic extension mechanism makes it both flexible and powerful. Specific components already available include: numerous data retrievers from our local databases; a chromosome map summarizing genes and genomic regions linked to CHDs; pie chart generation; gene network visualization and exploration; and candidate gene prioritization. This variety of components illustrates the versatility of the approach. For instance, pie charts are easily included by calling the lightweight pieChart component with the list of slices (name and value pairs), while the prioritization component consists in a complete web application specifically tuned towards prioritizing CHD genes. More generally, the generic extension proved successful in the fast development of new components working as wrappers for databases, web services, command line tools, and DAS (Distributed Annotation System) servers [16]. Also, to speed up the development time for structured data updates and interaction, the extension implements a generic mechanism to easily specify web forms in Wiki text that are pre-filled and handled by registered components.

In addition, CHDWiki interacts with a patient data repository, CHDBench [15], for managing patient data published in the literature, and a DAS server [17] feeding CHD genes and genomic regions has been set up to allow one to loop from CHDWiki to the Ensembl genome browser and back.

When more Wiki portals such as CHDWiki are available, the problems of interoperability of these systems and integration of stored knowledge can be managed through standard protocols such as DAS for data access, web services for the programmatic use of knowledge, or dedicated application programming interfaces (APIs), which will have to be further specified by the community. The component-oriented architecture of CHDWiki will make such future developments easy to implement.

The authors had full access to the data and take responsibility for its integrity.

## Results

### Overview of CHD data

**Figure 1:**
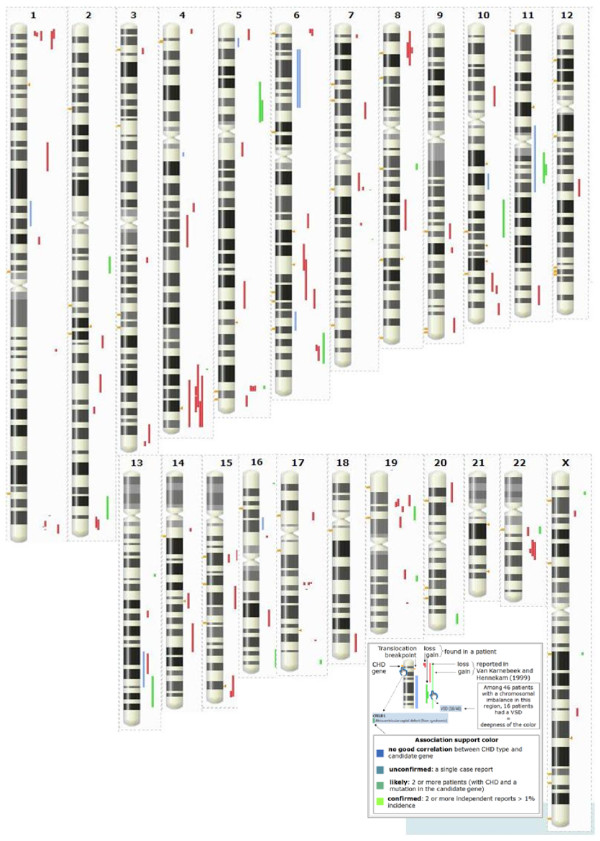
**Overview of CHD data plotted on a human karyogram**. A dynamic and interactive version is available at CHD:Map [32].

The results of the knowledge acquisition are described in Tables 1 and 2, and are visualized on an interactive chromosomal map in CHDWiki (Figure 1). They represent a unique repository of human genetic data for CHDs that describes both the phenotype and the genetic lesion with a granularity of detail that was unavailable so far. It allows for the addition of a free text description of any aspects of the gene that the contributor considers relevant.

**Table 1:** Number of features (genes, congenital heart defects, and so on) present in CHDWiki

Features	Entries
Genes	62
Congenital Heart Defects	83
Linkage regions	13
Balanced chromosomal aberrations	19
Van Karnebeek and Hennekam 1999 regions	46
Indel patients	155
Indel regions	176
References	198

**Table 2:** Number of relations between congenital heart defects (CHD) and currently managed features

Features	CHD
Genes	193
Linkage regions	14
Balanced chromosomal aberrations	21
Van Karnebeek and Hennekam 1999 regions	85
Patients	281
Studies (mutation screens)	297
Mutations	284

**Figure 2:**
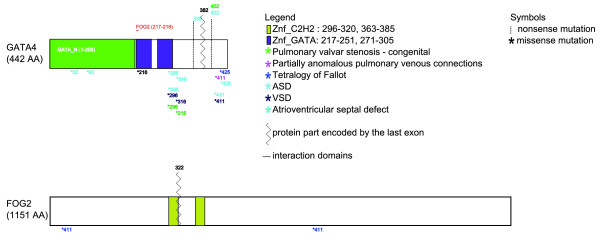
**Graphical overview of the encoded mutations and protein interaction domains of FOG2 and GATA4**. This map is designed to find possible associations between a cardiac phenotype and the mutational position within a protein domain. ASD, atrial septal defect; VSD, ventricular septal defect.

For each gene or phenotype, CHDWiki provides a pie diagram that graphically represents the spectrum of related cardiac phenotypes or mutated genes, followed by a detailed overview of the studies defining the mutational spectrum of these gene-phenotype links. Moreover, to get an intuitive view of the mutation data, a graphical map of all proteins is automatically produced and updated as new genotypes are entered, displaying the position of coding mutations in the context of the protein domains. The relevance of this display is highlighted by the significant clustering of missense mutations in annotated domains (Additional file 1). More features could be added in the future (sites of protein-protein interaction or post-translational modification, for example, Figure 2).

Apart from mutations detected at the nucleotide level, CHDWiki readily incorporates copy number variations and other disease-linked chromosome anomalies. Such 'chromosomal mutations' have recently been shown to be important in many disorders, including CHDs [18,19].

Additionally, this portal offers a graphical overview of the protein interaction partners, as well as external links to both human and non-human genome browsers and model organism databases. Moreover, via an automated text-mining approach, genes potentially implicated in a given CHD and, vice versa, CHDs caused by a gene of interest are returned [20,21]. Finally, to help researchers to select candidate genes for CHDs in sets of genes (for example, identified in regions of the genome that are found through linkage analysis, homozygosity mapping or chromosomal aberrations), an adapted Endeavour algorithm for gene prioritization was implemented in CHDWiki. It offers predefined training sets of genes with tailored data sources (further details at [22]).

### Synthetic graphical data representation

**Figure 3:**
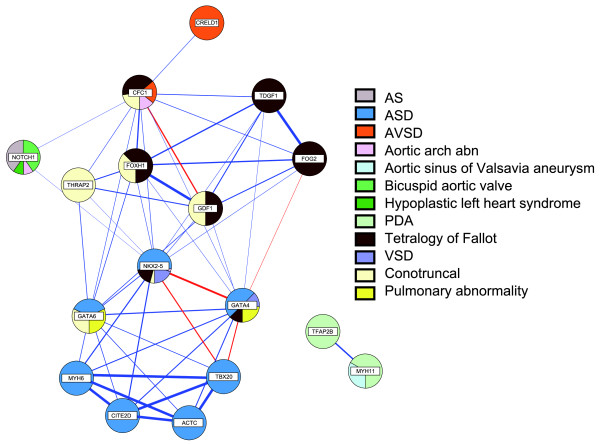
**Network of genes (nodes) sharing non-syndromic cardiac phenotypes when mutated (edges)**. The nodes are represented as pie charts displaying gene-linked CHD types as well as their frequency. Unconfirmed gene-phenotype relations based on single case reports were not included. The width of the edges (log of euclidian distance) depends on the number and the percentage of shared phenotypes. Known protein-protein interactions are represented by red edges. Proteins sharing multiple phenotypes when mutated tend to act in the same molecular pathways or even encode proteins that directly interact (for example, TBX20 physically interacts with NKX2.5 and GATA4 [33]. By further expanding the database, phenotype sharing will enable us to predict novel protein interaction partners. On the other hand, we hypothesize that further insights into the molecular basis of the developing heart will point towards novel candidate genes for a specific CHD type based upon the phenotypic spectrum of a known interaction partner. For example, since mutations in *CFC1 *are associated with laterality defects and conotruncal heart defects, other players in the NODAL signaling pathway (*FOXH1*, *TDGF1 *and *GDF1*) were considered to be likely candidates for these CHDs [34,35]. AS, aortic stenosis; ASD, atrials septal defect; AVSD, atrioventricular septal defect; PDA, patent ductus arteriosus; VSD, ventricular septal defect.

Associations between genes and phenotypes can be converted to networks and visualized as such. As CHDWiki is very detailed, additional features can be added to such a network (Figure 3), that is, specification of the observed gene-linked phenotypes, their frequency, the number of phenotypes shared by two genes and physical protein-protein interactions. Figure 3 is thus a synthetic representation of all data on non-syndromic CHDs. We also built similar networks for syndromic genes (Additional file 2) and networks where diseases are connected when caused by the same genes [23].

## Discussion

### Wiki portals

We argue that, next to the development of a Wikipedia for Genes, another major application of Wiki-like collaborative technology is the development of Wiki knowledge and analysis portals. In many research areas, the body of common knowledge is usually scattered across reviews, original articles, and genomic databases (MEDLINE, Ensembl and UCSC genome browsers, Entrez, OMIM, and many others). A structured Wiki allows the integration of all these data into a view that is centered on the needs of a community. A large number of bioinformatics analysis methods are available, yet a significant gap remains towards their integration within the daily practice of most biologists. By tightly integrating a knowledge base with dedicated analysis tools, a Wiki portal provides a natural stepping stone for biologists to use advanced data analysis techniques. While the development of data analysis portals has been a long-standing aim of bioinformatics, few convincing applications with a clear impact on biology have been demonstrated since the development of homology-based methods [24,25]. We believe that the causes of this lack of progress are mainly a lack of fine-tuning to the needs of a specific biological research area and the complexity of data analysis strategies, which appear overwhelming to biologists. We believe that Wiki portals alleviate those problems in several ways: first, by lowering the threshold towards data analysis by a tight integration with a knowledge base that is immediately useful to any biologist from the community; and second, through development of data analysis strategies sufficiently user friendly to be within reach of the majority of biologists (such as prioritization strategies, network browsing, and so on).

### Impact on knowledge acquisition and exchange

In the presented CHDWiki, we compiled all information available in the literature on CHD genetics to construct a collaborative portal. We thus introduce the first example of a new type of literature review, which is dynamic and evolving; it readily provides researchers, geneticists, physicists and cardiologists with direct access to the most comprehensive knowledge on the genetics of a particular disease. An immediate impact of this methodology is how it complements and enhances the process of reviewing the literature. Wikis are clearly dynamic in nature, and can accompany a review article so that information can be kept up-to-date after the publication of the original review. A second advantage is that they cross-link interpretation and actual data: browsing such a review provides a straight link into relevant data sources (genome browsers, publications, and so on). Querying the genome on the other hand directly links into an integrated and up-to-date review. A third advantage is obviously their collaborative nature, where the expertise of numerous researchers can be pooled together to obtain extensive peer-reviewed knowledge.

The impact of disease-oriented Wikis is quite significant for both clinicians and researchers. For clinicians, it allows them to find out instantly if a gene or region has been linked to the phenotype of their patients. For instance, the finding that a chromosomal imbalance in a CHD patient affects an annotated CHD gene or region provides conclusive evidence for its causality, thus facilitating diagnosis and therapy. For the researcher, several novel regions recurrently linked to CHDs in patients emerge from the compiled genomic data, such as 1p36.3, 6q21, 15q26, 16p13.3, 17q21.3, and 22q12 (Figure 1). These provide an entry point to try to identify novel genes linked to human CHDs as illustrated [22]. Further studies are underway to obtain conclusive evidence for the involvement of these genes in CHDs, but their potential involvement is readily annotated in CHDWiki and is available to the community.

Specialized Wiki portals like CHDWiki may play a key role in the development of a universal 'Wikipedia of Genes'. Content from specialized Wikis can be compiled into a Gene Wikipedia. There might be some challenges with such integration, so an alternative model would be a federation of Wikis, where no centralized Gene Wikipedia exists, but where a hub/search engine passes queries to registered Wikis and the results are aggregated. The latter solution seems more likely since it is more efficient to develop a specialized Wiki portal, such as CHDWiki, providing specific tools for the community and focusing on a particular area. For instance, the Wikipedia page on Tetralogy of Fallot is directed towards a large audience with sections such as symptoms, diagnosis and treatments, while the CHDWiki page describes this phenotype succinctly and provides extensive knowledge on the genetics of this CHD (genes and mutations, patient reports). Another example is from Wikiproteins, which aims at being exhaustive about available protein knowledge, where the GATA4 entry currently links to several species and provides general information (function, localization, structure) but where its link to CHDs (ASD, Tetralogy of Fallot, and so on) is briefly and incompletely mentioned.

Wiki portals also perfectly fit with the philosophy of DASs. Not only can the data of any gene - or genomic feature - be distributed among portals and collaboratively annotated, but the genome browser can also serve as an entry point to the Wikis. CHD researchers who enable the CHDWiki tracks in a genome browser directly visualize any information compiled in CHDWiki.

### Collaborative aspects

An important asset of specialized Wiki portals is that they could be a solution to the long-term maintenance of biological knowledge bases. Such long-term maintenance is a major recurring issue for the biological and bioinformatics community because of the persistent lack of long-term funding. We envisage a model for community-driven knowledge bases where the original portal development is funded through a classical grant, but where the curation of the knowledge base is gradually shifted from the core development team to the community at large over the period of the grant (3 to 5 years). Data curation should be included in the original development cycle until a critical mass of information is reached that guarantees adoption by the community, as has been done for CHDWiki (described in the knowledge acquisition section). After curation has been shifted to the community, technical portal maintenance can be minimal.

Although such collaborative tools can easily exchange information and queries over the web, there would remain a significant risk of fragmentation if no protocols and strategies were available to effectively exchange information among the myriad of tools that are currently emerging (that is, we would have a Tower of Babel of internet-enabled tools). To satisfy the need of standardized and unified web accessible databases allowing simple data exchange, several bioinformatics initiatives have recently emerged promoting the Semantic Web (Concept Web Alliance, HCLS [26]). Indeed, the Semantic Web offers a general format for data interchange, thus providing curators with a standardized framework allowing data to be integrated and reused across disciplines.

In biology, the advantages of the Semantic Web are obvious: unique names for biological entities and consistent standards for knowledge representation, retrieval, and processing. Such standards simplify integration of web resources as they can be queried the same way. Bioinformatics query systems in diverse fields have started to use this technology: examples include WikiPathways, a wiki dedicated to the precise collaborative annotation of metabolic pathways [27], integration of ALFRED (an allele frequency database) [28], and cancer pathways of the Starpath resource [29], and so on.

Although CHDWiki is not directly compatible with the Semantic Web approaches, it was implemented using a standard nomenclature for all terms (for example, AEPC terms for disease, EnsEMBL identifiers for genes), thus allowing straightforward transfer of its content to the new semantic web standards (for example, GEN2PHEN, a resource aimed at unifying genetic variation databases). Moreover, the CHDWiki DAS server already allows users to integrate the CHDWiki data in the EnsEMBL genome server. An important further development of our platform in this respect will be the implementation of web services following current semantic web standards. Indeed, some recent initiatives (for example, Semantic Web Mediawiki) now allow developers to combine Wiki and semantic web technologies.

Wikis also provide an effective solution to the enduring problem of unpublishable or negative results (for example, of mutation analyses). These can be highly valuable for other research teams pursuing similar paths of investigation, or contain relevant information below statistical significance. Wiki portals like CHDWiki are a natural repository for such findings. Also classic collaborative features, such as a mailing list (for example, for collaboration requests, job postings, event announcements and exchange of biological material such as DNA, cell lines, or cardiac tissue) and a directory of researchers, are basic but valuable tools such a portal can offer.

### Outstanding issues

A number of technical and social issues may require significant further technology developments. The first is access control. Given the functionalities provided by a Wiki portal, it comes close to a collaborative laboratory notebook. However, research teams will understandably be reluctant to share their experimental data prior to publication. Access control restrictions (that is, deciding to provide access to part of the information to only a selected group of people) will require some further development of the Wiki platforms, although this is basically a technical problem that is routinely solved in database systems.

A second issue is quality control. Wikipedia is based on a model of peer-editing, which is generally effective but can sometimes lead to conflicts between contributors (edit wars). Such conflicts are likely to arise when conflicting scientific hypotheses and interpretations collide. Additionally, conflicts are also likely regarding scientific attribution and priority. For smaller Wikis, a 'benevolent dictator' who oversees any conflict can solve those problems. For larger Wikis, a hierarchy of editorial roles (contributor, reviewer, and technical, associate, and executive editors) might become necessary.

A third issue is credit assignment. Scientific credit is an essential driver and one cannot expect researchers to contribute a significant proportion of their time if no credit is attributed to them. For smaller Wikis, citation of a key publication describing the Wiki will be sufficient (exactly like for specialized databases) because key contributors will be among the authors. For larger Wikis, a direct crediting system might be difficult to establish. Significant contributions to Wikis (as contributor or editor) should be recognized as relevant scientific work and therefore appear in the track record of a researcher. Related to the issue of credit is the issue of citation. As some Wiki entries may become key scholarly references, proper citation to accurate dates or versions might be needed in scholarly work because entries change over time.

Regarding software development, each knowledge portal is likely to have highly specific aspects (type of information, source databases, ontologies or analysis tools) that make the development of a single off-the-shelf solution (similar to MediaWiki and other Wiki software) highly unlikely. A more realistic solution will lie in the development of generic tools that enable the flexible construction of such Wikis by embedding generic modules for ontology management, XML data representation, visualization, database query, and data analysis. The goal of such software will be to maximally speed up the development of Wikis while minimizing functional constraints. The current version of our Wiki framework has been made available as an open source project [30].

## Conclusions

The future we envisage is one where a specialized community 'swarms' around a Wiki portal that provides most of the knowledge, data, and analysis tools needed to support its experimental work. Via this portal, the community can collaboratively and incrementally chart complex networks involved in biological processes, phenotypes, and diseases. Collaboration and efficient access to knowledge, data, and tools will significantly speed up experimental research.

## Availability and requirements

CHDWiki is accessible at [31]. Data can be consulted without any registration; however, to add or modify Wiki information an account must be requested by clicking on the 'log in/create account' button at the top right of the main page.

## Abbreviations

AEPC: Association for European Paediatric Cardiology; CHD: congenital heart defect; DAS: Distributed Annotation System.

## Competing interests

The authors declare that they have no competing interests.

## Authors' contributions

RB, JB, BT and SB participated in the design of the study and drafted the manuscript. KD and YM conceived of the study, participated in its design and coordination, and drafted the manuscript. All co-authors contributed to various aspects of the platform development: Wiki development (RB, SB, YM), Gene networks (RB, SB), genotype-phenotype relations (JB, BT, KD), Bench (SVV, BC), and gene prioritization tools (LCT, PVL). Knowledge acquisition was performed by JB, BT, KD and MG. All the co-authors listed above fulfill the three requirements for authorship defined by the ICMJE guidelines.

## Supplementary Material

Additional file 1 A figure of nonsense mutations encoded in the CHDWiki for the proteins NKX2-5 and TBX5. Displayed here are mutations in NKX2-5 and TBX5 present in CHDWiki. Missense mutations (asterisks) are significantly enriched in functional domains (*P*-values: NKX2-5, 1 × 10^-3^; TBX5, 0.05; across all nonsydromic genes, 1 × 10^-4^). This finding is independent of the ascertainment bias associated with preferential classification of mutations affecting protein domains as pathogenic: missense mutations identified through linkage analysis in multiple individuals similarly affect preferentially protein domains (*P*-values: NKX2-5, 0.02; TBX5, 0.05; all nonsyndromic genes combined, 0.03). This graphical representation moreover enables straightforward genotype-phenotype correlations: missense mutations causing atrial septal defects are preferentially affecting the homeobox domain (*P*-value: 1 × 10^-4^). Click here for file

Additional file 2 A figure showing a network with genes sharing syndromic cardiac phenotypes when mutated. Network with genes (nodes) sharing syndromic cardiac phenotypes when mutated (edges). As members of the RAS-MAP kinase pathway (PTPN11, SOS1, BRAF, KRAS, MAP2K1, MAP2K2, SHOC2 and NF1) clearly form a phenotypic cluster, they seem to be involved in the same developmental cardiac cell lineages. Click here for file
