# Supplementary figures and images for: Collaboratively charting the gene-to-phenotype network of human congenital heart defects
Source: Genome Med. 2010 Mar 1;2(3):16. doi: 10.1186/gm137 (PMC2873794; doi:10.1186/gm137)

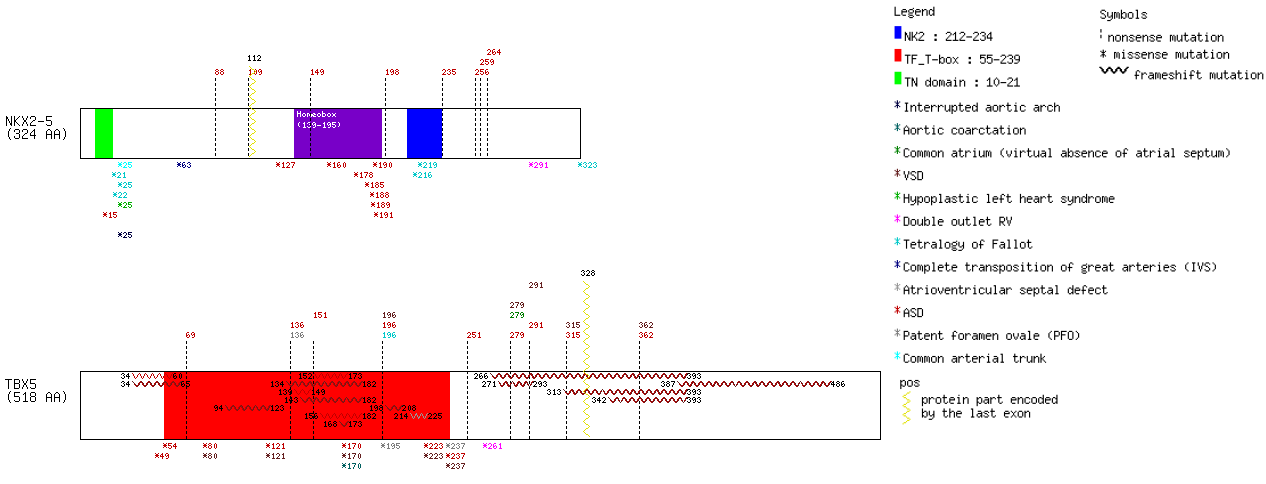

Supplement: Additional file 1 — A figure of nonsense mutations encoded in the CHDWiki for the proteins NKX2-5 and TBX5. Displayed here are mutations in NKX2-5 and TBX5 present in CHDWiki. Missense mutations (asterisks) are significantly enriched in functional domains (P-values: NKX2-5, 1 × 10^-3^; TBX5, 0.05; across all nonsydromic genes, 1 × 10^-4^). This finding is independent of the ascertainment bias associated with preferential classification of mutations affecting protein domains as pathogenic: missense mutations identified through linkage analysis in multiple individuals similarly affect preferentially protein domains (P-values: NKX2-5, 0.02; TBX5, 0.05; all nonsyndromic genes combined, 0.03). This graphical representation moreover enables straightforward genotype-phenotype correlations: missense mutations causing atrial septal defects are preferentially affecting the homeobox domain (P-value: 1 × 10^-4^). [file gm137-S1.TIFF]
